# Yu ping feng san for pediatric allergic rhinitis: A systematic review and meta-analysis of randomized controlled trials

**DOI:** 10.1097/MD.0000000000024534

**Published:** 2021-04-02

**Authors:** Yong Liao, Juan Zhong, Shuqin Liu, Menglin Dai, Yang Liu, Xinrong Li, Yepeng Yang, Dazheng Zhang, Dan Lai, Tao Lu, Qinxiu Zhang, Yu Zhao

**Affiliations:** a Department of Otorhinolaryngology Head and Neck Surgery, West China Hospital of Sichuan University, Chengdu; b Department of Otorhinolaryngology Head and Neck Surgery, Minda Hospital of Hubei Minzu University, Enshi; c Hospital of Chengdu University of Traditional Chinese Medicine; d School of Medical and Life Sciences/Reproductive and Women-Children Hospital, Chengdu University of Traditional Chinese Medicine; e Chengdu University of Traditional Chinese Medicine; f Dujiangyan medical centre; g China qingcheng medical research laboratory of Traditional Chinese Medicine; h Department of Otolaryngology Head and Neck Surgery, The Affiliated Hospital of Southwest Medical University, No. 25 TaiPing Street, Luzhou, Sichuan, China; i Otolaryngology and Head & Neck Surgery Department One, First Affiliated Hospital of Kunming Medical University, Kunming, Yunnan.

**Keywords:** allergic rhinitis, systematic review, Yu ping feng san

## Abstract

**Background:**

The potential treatment effects and safety of Yu ping feng san (YPFS) for pediatric allergic rhinitis (PAR) patients have yet to be studied systematically.

**Objectives:**

To assess the effects and safety of YPFS for treat pediatric patients, allergic rhinitis.

**Methods:**

We systematically searched PubMed, EMBASE (Excerpta Medical Database), Cochrane library, Chinese Cochrane Centre's Controlled Trials Register platform, Wanfang Chinese Digital Periodical and Conference Database, China National Knowledge Infrastructure Database, and VIP Chinese Science, from inception dates to November 1, 2019. Randomized controlled trials (RCTs) were included. The risk of bias in the trials was assessed in accordance with the Cochrane Handbook, version 5.1.0. RevMan 5.3 software was used to perform a meta-analysis. Grading of Recommendations Assessment, Development and Evaluation methodology was applied to evaluate the evidence quality for each outcome. The quality of evidence for each outcome measurement was low for 4 outcomes and very low for 5 outcomes.

**Results:**

A total of 10 RCTs involving 1069 participants (3–15 years old) fulfilled the inclusion criteria. After exclusion, 8 RCTs were pooled for efficacy assessment. The overall efficacy evaluation result did not show benefit for the experimental group (relative risk 0.32, CI 95% 0.24–0.45; *P* = .98;) Investigation of variation of serum IgA, immunoglobulin E, IgG in three studies in 2 groups returned no statistical significance. YPFS gave relatively better safety (relative risk 0.29, CI 95% 0.14–0.58; *P* = .0005; Fig. S8 and lower recurrence rates than did Western medical therapy.

**Conclusions:**

Current evidence cannot support the routine use of YPFS for treatment of PAR. This may be due to poor-quality study-design limitations of the included YPFS studies. Our data showed that the use of YPFS for PAR is relatively safe compared to Western medical therapy, but a conclusion could not be drawn because only 5 studies were analyzed. Every study suffered from some methodological limitation. Therefore, further large, rigorously-designed studies are necessary to determine conclusively the utility of YPFS in PAR.

## 1 Introduction

Allergic rhinitis (AR) is a chronic inflammation of the nasal mucous membranes, thought to result from immunoglobulin E-(IgE)-mediated sensitization to environmental allergens, including dust, domestic animals, pollens, and molds. AR is characterized by the onset 2 or more of the following nasal symptoms: nasal discharge, sneezing, nasal itching and congestion, all of which interfere with activities of daily living and incur substantial social economic burdens. The incidence of pediatric allergic rhinitis (PAR) in China was approximately 8% to 24.1% in 2014.^[1]^ A review of the Chinese pediatric population showed that the comorbidity of PAR was high in Chinese children in 2018, especially because AR often accompanies asthma. The incidence of asthma in children with AR is 35.01% and the incidence of AR in children with asthma is 54.93%,^[2]^ suggesting that controlling the disease is critical. Oral antihistamines and immunotherapy are recommended by a guideline for AR.^[3]^ Chinese herbal medicine for management AR in clinical practice is mentioned in this guideline, however, the evidence for herbal medicine for AR remains uncertain. Yu ping feng san (YPFS) formula, is composed of three herbs: Huang Qi (*Astragalus membranaceus*), Bai Zhu (*Rhizoma atractylodis macrocephalae*) and FangFeng (*Radix ledebouriellae divaricatae*). YPFS and Yu Ping Feng Decoction (YPFD) are both composed of three herbs: Huang Qi (*Astragalus membranaceus*), Bai Zhu (*Rhizoma atractylodis macrocephalae*) and FangFeng (*Radix ledebouriellae divaricatae*). These are used for treatment of “deficiency syndromes.”They are 2 types of 1 formula. In Traditional Chinese Medicine theory, the differences between YPFS and YPFD lie in the dosage form and usage method. YPFS is a powder formula for oral taking after stirring it while YPFD is a water decoction formula. Patients use it by cooking and drinking the juice. Their routes of administration are the same in modern Chinese Medicine; that is, by cooking and drinking the juice. Hence, YPFS can be viewed as YPFD in clinical use.

The decoction was recommended by Chinese medicine clinical practice guidelines for management of AR despite its therapeutic mechanisms not being clear.^[4,5]^ According to Traditional Chinese Medicine theory, the pathogenesis of AR falls into 1 of 2 categories: excess or deficiency syndromes. YPFS is thought to act on “deficiency syndromes” by strengthening bodily resistance. A number of clinical studies^[6,7,8]^ on YPFS for treatment AR or PAR had been performed in recent years. However, none of these includes a review of YPFS as experimental intervention for PAR. Hence there is an urgent need for a systematic review to summarize the evidence from all available studies of YPFS for PAR. The purpose of this review was to assess critically the current state of evidence from randomized controlled trials (RCTs) on the use of YPFS in PAR according to the Cochrane Handbook of meta-analysis.

## 2 Methods

### 2.1 Search strategy

A systematic search for YPFS for PAR trials was performed on November 1, 2019. All published and ongoing RCTs were searched. The languages were limited to Chinese or English.

### 2.2 Electronic searches

A total of 6 databases were searched, including PubMed (1992 to November 1, 2019), EMBASE (Excerpta Medical Database) (1992 to November 1,2019), Cochrane Library (Issue 9 of November 1, 2019), Chinese Cochrane Centre's Controlled Trials Register platform (up to November 1, 2019), Wanfang Chinese Digital Periodical and Conference Database (1997 to November 1, 2019), China National Knowledge Infrastructure Database (1992 to November 1, 2019), and VIP Chinese Science and Technique Journals Database (1992 to November 1, 2019). In addition, the Chinese Clinical Trial Registry Center was searched retrieved for ongoing trials.

Search terms were as follows:

#1 Yu ping feng powder
#2 Yu ping feng san
#3 YPFP
#4 YPFS
#5 Chinese Herbal Medicine
#6 integrated Chinese and Western medicines
#7 integrated traditional and Western medicine
#8 #1-#7/OR
#9 pediatric allergic rhinitis
#10 pediatric rhinallergosis
#11 PAR
#12 #9-#11/OR
#13 #8AND#12

### 2.3 Other search resources

In order to ensure complete searches for all trials, we checked references of related identified publications.

### 2.4 Study types

Prospective RCTs of YPFS versus placebo, or conventional medicine were included in this review. Studies were included if YPFS combined with Western medicine therapy as experimental intervention or if identical Western medicine therapy was the control group intervention. However, if another Chinese Herbal Medicine formula or another Traditional Chinese medical rehabilitation method was studied, including acupuncture, cupping and moxibustion therapy combined with YPFS as experimental invention, the study was not included due to the difficulty in evaluating the effectiveness of YPFS in this model. Observational studies, case reports, case series, qualitative studies, uncontrolled studies, and studies with no real randomization-control design were excluded.

### 2.5 Participants

Patients presenting with seasonal PAR or perennial PAR were all included. Taking into consideration the fact that targeted drug combination methods could not be used to compare effects, trials involving PAR combined with pediatric allergic asthma or pediatric allergic conjunctivitis and other allergic diseases were excluded.

### 2.6 Interventions

We compared YPFS with conventional medicine or placebo regimens. Interventions considered for experimental groups vs control groups were as follows:

(1) YPFS vs conventional medicine, including fluticasone propionate nasal spray, desloratadine tablets, Singulair, oxymetazoline hydrochloride nasal spray, cetirizine and budesonide nasal aerosol.
(2) YPFS combined with conventional medicine vs conventional medicine.
(3) YPFS vs placebo.

The form of YPFS either powder or decoction or granules were all included.

### 2.7 Outcome measures

Trials were required to include as outcome measures either relief of PAR symptoms or assessment of the efficacy of YPFS for PAR. Other important clinical outcomes included recurrence rate, serum IgE or IgA or IgG level, improvement in symptom scoring, and adverse events.

The efficacy of YPFS for PAR, and improvement of symptom scoring were set as primary outcomes. Serum IgE or IgA or IgG level, recurrence rate and adverse events (including dry mouth, headache, and gastrointestinal discomfort) were set as secondary outcomes.

### 2.8 Study selection, data extraction, and quality assessment

#### 2.8.1 Study selection

After three authors (YL, JZ, SQL) scanned all titles and abstracts, a judgment was made regarding whether the trials met our inclusion criteria. Full-text screening was the next step, accomplished by 4 authors (DL, XRL, DDZ, TL). If there were conflicts, they were resolved by consensus.

#### 2.8.2 Data extraction and management

Raw data of included papers included study ID, details regarding first authors and publication years, design details of the original study (duration, diagnostic criteria, efficacy assessment criteria, interventions for 2 groups, period, outcome measures, balance report of baseline, and randomization method). These were separately extracted by three authors (YL, YZ, JZ).

### 2.9 Assessment of risk of bias

The methodological quality for the included RCTs was assessed based on the Cochrane Handbook for Systematic of Interventions. The latest version of this tool was updated in November 2011, version 5.1.0 (http://www.handbook.cochrane.org).^[9]^ Three authors (YL, LL, YZ) performed this analysis. Risk of bias items included the following: randomization sequence generation, allocation concealment, blinding of participants or healthcare providers, detection bias, incompleteness bias, reporting bias, and other bias. We defined other bias as trials may be sponsored by drug experts, in whose trials baseline characteristics were not similar between different intervention groups. We assessed publication bias by examining funnel plots when the number of trials reporting the primary outcomes was 10 or more.

### 2.10 Statistical analysis

Review Manager (RevMan) software version 5.3 was applied to pool our data and to execute the meta-analysis. Grading of Recommendations Assessment, Development and Evaluation (GRADE) software was the sole quality evaluation tool used to demonstrate the GRADE evidence ratings.

Risk ratio (RR) was chosen for dichotomous data (efficacy, recurrence rate, and adverse events). Mean difference was chosen for variable data. Confidence interval (CI) was set at 95%, and *P* < .05 was defined as statistically significant. *I*^2^ values were used to assess inter-study heterogeneity. According to the Cochrane Handbook, when *I*^2^ > 75%, considerable heterogeneity was confirmed, whereupon a random effects model was applied. We pooled trials when the intervention form of those studies was adequately similar. Specific subgroups were analyzed according to similar intervention forms or similar design. Based on the practice recommendation of the Cochrane Handbook, we found that zero events in both the intervention and the control groups could be excluded from the meta-analysis.

## 3 Results

### 3.1 Study description

#### 3.1.1 Search results

One hundred and sixty-eight trials were identified by us initially according to our protocol search strategy. No unpublished or ongoing studies were found. After titles, abstracts, and keywords were reviewed, 160 papers were excluded for failure to conform to inclusion criteria. Twenty-six duplicated texts were excluded, as were 13 studies that had initially appeared to meet our inclusion criteria. After the full texts were read, three studies were excluded because of absence of diagnostic criteria,^[10]^ or semi-randomization methods.^[11]^ In another, the herbal medicine formula Cang Er Zi San was included as the treatment group intervention.^[12]^ Ten studies finally met our inclusion criteria. The study selection process is outlined in Supplemental Digital Content Figure 1

### 3.2 Included studies and excluded studies

There were commonly 10 Chinese language trials containing1069 pediatric participants aged 3 to 15 years old^[13–22]^ included and pooled in our review. Those trials were published between 2006 and 2017. According similar intervention type, we divided these 10 studies into three subgroups: Two studies^[18,19]^ with pattern of YPFS vs. Western medicine therapy (WM); three^[17,21,22]^ studies with the mode of YPFD+WM vs. WM; the remaining 5 studies^[13–16,20]^ used YPFS+WM vs WM intervention design. Based on consistency of measurement on effective rate outcome in all of 10 studies, the effective rate indicator was regarded as the most important outcome measure in all trials. There were totally 6 trials^[14–16,19–21]^ recording adverse events, and 2 studies^[17,19]^ reported recurrence rate. One study^[15]^ reported levels of serum IgA, IgG, IgE, IL-6, IL-17 and IL-23 before and after treatment. Another study^[13]^ recorded serum IgA, IgG, IgE, PO_2_, PCO_2_ and WBC value as well. One study^[21]^ reported time required for olfactory recovery, disappearance of nasal mucosa edema, and patient satisfaction with disease management. In 1 paper published in 2015,^[20]^ clinical symptom score, clinical signs score and variation of serum IgA, IgG, IgE were reported.

No participant withdrawal information was directly reported in these studies. No indicator of the influence on life quality as an outcome measure was reported in those studies. Characteristics of 6 included studies are displayed in Supplemental Digital Content Table 1.

In Ye (2014),^[11]^ all participants were allocated to experimental and control groups by visit sequence. Thus, this study could be defined as a semi-randomized trial. In another article published in 2014,^[12]^ the Chinese herbal medicine Cang Er Zi San preparation was combined into the treatment group intervention. This kind of comparison does not conform to the inclusion criteria. In the third study published in 2017,^[10]^ there was lack of reliable diagnostic criteria. Therefore, these three studies were excluded. Characteristics of the three excluded studies are displayed in Supplemental Digital Content Table 2.

### 3.3 Risk of bias

#### 3.3.1 Allocation (selection bias)

Ten studies were designed as RCTs, of which 2 studies^[15,20]^ randomly divided all participants using a random number table tool. However, the authors of these studies failed to report details of the patient allocation technique. Hence, a high risk of bias could be ascribed to these trials. The randomization methods and allocation concealment details of patient distribution in the remaining studies were not mentioned in the original texts. Therefore, there may be a high risk of selection bias in these trials.

### 3.4 Blinding (performance bias and detection bias)

No blinding method was mentioned in any of the 10 included studies. Thus might present a high risk of performance and detection bias.

### 3.5 Incomplete outcome data (attrition bias)

None of the included studies reported information regarding sample size calculation. No study directly provided information regarding cases lost to follow-up or study withdrawals. However, in Yang's^[19]^ study, there was information regarding dropout from follow-up; thus only 11 patients in the treatment group and 6 in the control group participated in the follow-up. Therefore, the risk of incomplete outcome data bias in this study was low and in others it was unclear.

### 3.6 Selective reporting (reporting bias)

None of the 10 included trials noted the protocol, and none of the trials declared a clinical trial registration number. However, in a study written by Fang in 2017,^[15]^ the author noted that their trial gained ethics association approval, and all patients gave written informed consent before the experiment. According to regulations of local ethics committee, there would have to have been an advanced approval protocol. Therefore, selective reporting bias in this study was low and in others it was unclear.

### 3.7 Other potential sources of bias

Although no signs of pharmaceutical company support were found in these studies, 1 study by Xu^[18]^ made no reference to balanced report at baseline. Therefore, other bias of this paper was high and in others it was low.

The risk of bias graph is displayed in Supplemental Digital Content Figure 2 and the risk of bias summary is displayed in Supplemental Digital Content Figure 3.

### 3.8 Effects of interventions

Ten studies comprising 1069 pediatric participants, were included in this review. However, in the studies of Fan^[14]^ and Li,^[16]^ there were zero experimental event, therefore these 2 could not be pooled. Intervention forms used in RCTs can be classified into three types. Therefore, we performed intervention-specific sub-analyses of RCTs according to the three types of intervention measure. Variation of serum IgA, IgE and IgG were evaluated. Adverse events (safety) and recurrence of YPFS for AR were also analyzed.

The efficacy of 8 RCTs comparing particular interventions and YPFS is presented in Supplemental Digital Content Figure 4 Variation of serum IgA is presented in Supplemental Digital Content Figure 5 Variation of serum IgE is presented in Supplemental Digital Content Figure 6 Variation of serum IgG is presented in Supplemental Digital Content Figure 7 Adverse events (safety) evaluation of YPFS is presented in Supplemental Digital Content Figure 8.

### 3.9 YPFD+WM vs. WM (three RCTs)

Three RCTs^[17,21,22]^ tested the efficacy of YPFD combined with Western medicine compared with Western medicine alone. We pooled these trials using RevMan 5.3. YPFS was not superior to Western medicine therapy (RR 0.35, CI 95% 0.20–0.61; *P* = 0.89; Supplemental Digital Content Figure 4).

### 3.10 YPFS+WM vs. WM (Three RCTs)

Three clinical trials^[13,15,20]^ assessed the effectiveness of YPFS+WM vs. WM pattern. The result suggests an advantage for WM over YPFS combined with WM (RR 0.29, CI 95% 0.18–0.48, *P* = 0.64; Supplemental Digital Content Figure 4).

### 3.11 YPFS vs. WM (2 RCTs)

Two trials^[18,19]^ made this mode of comparison. The control group intervention had a better treatment effect than did the YPFS group (RR 0.34, CI 95% 0.18–0.64; P = 0.60; Figure 4). The overall effect was not good for the experimental group (RR 0.32, CI 95% 0.24–0.45; P = 0.98; Supplemental Digital Content Figure 4).

### 3.12 Variation of serum IgA, IgE, IgG

Three studies^[13,15,20]^ reported variation in serum IgA, IgE and IgG before and after treatment, totaling 452 participants in every comparison. We pooled these trials using RevMan 5.3. *I*^2^ value was equal or greater than 70 percent in these comparisons. The heterogeneity in every comparison therefore differed widely. No comparisons showed significant differences between 2 groups on the basis of the point of intersection in every forest plot (Supplemental Digital Content Figure 5, Supplemental Digital Content Figure 6, Supplemental Digital Content Figure 7). Thus, variation of serum IgA, IgE, IgG between groups showed no significant difference.

### 3.13 Adverse events

Six studies^[14–16,19–21]^ reported the details of adverse events. Li^[16]^ and Fan^[14]^ reported no adverse events in either group, therefore, these results were not be pooled. Fang^[15]^ reported adverse events in 4 participants (13.33%; 1 sleepiness, 2 headache and 1 gastrointestinal discomfort) in the experimental group, while in the control group, 10 participants had adverse events (33.33%; 2 sleepiness, 4 headache, 2 gastrointestinal discomfort and 2 dryness of the mouth). Those symptoms abated when the patient stopped the medications. Yu2015^[20]^ reported adverse events in 7 participants in the experimental group (19.4%; 1 sleepiness, 1 fatigue, 1 gastrointestinal discomfort, 1 rash and 1 dryness of the mouth), while there were adverse events in 22 participants in the control group (61.1%; 6 sleepiness, 3 fatigue, eight gastrointestinal discomfort, 2 rash and three dryness of the mouth): X^2^ = 12.9912, *P* = .0003, with YPFS safer than WM in this study. Yu 2016^[21]^ reported an adverse event in 1 participant (3.3%; fatigue) in the experimental group, while there were 14 participants with adverse events in the control group (46.7%; 4 headache, 6 fatigue, and 4 dryness of the mouth). Yang2010^[19]^ reported 36 pediatric patients with adverse events (19 spontaneous sweating, 12 with poor appetite and 1 rash) in the control group after treatment, and three patients with adverse events (1 spontaneous sweating and 2 with poor appetite): YPFS was safer than the control group (*P* < .05). However, in this paper, the percentage was not calculated and there were 36 pediatric patients with adverse events in control group, greater than overall number of control group participants. It was possible that there were several events in 1 person; therefore, the result could not be pooled. We pooled the others, and the result was that YPFS was safer than WM (RR 0.29, CI 95% 0.14–0.58; *P* = .0005; Supplemental Digital Content Figure 8).

### 3.14 Recurrence rate

Two studies^[17,19]^ reported condition at follow-up visit. Lin^[17]^ noted that a month after medication withdrawal, 66.67% (37 participants) never relapsed in the experimental group, while 23.73% (14 participants) never relapsed in the control group (*P* < .01). In another trial, the author Yang^[19]^ reported that not all of patients took part in the 2-month follow-up. Sixteen patients (64%) participated: three (18.8%) had recurrence in experimental group, while 11 patients (52%) took part and 4 (36.4%) recurred in the control group. However, Yang did not make an intention to treat analysis to cases of depigmentation in his follow-up.

### 3.15 Quality of evidence

The quality of evidence for outcome measures according to the GRADE system is displayed in Supplemental Digital Content Tables 3, 4, 5, 6 and 7.

## 4 Discussion

### 4.1 Overview of findings

Although there is a review about Yu ping feng san for adult allergic rhinitis,^[23]^ reviews of Yu ping feng san for PAR have not been reported before. Therefore, to the best of our knowledge, this is the first meta-analysis of YPFS therapy for AR pediatric patients.

In allergic rhinitis, numerous inflammatory cells, including mast cells, CD4-positive T cells, B cells, macrophages, and eosinophils, infiltrate the nasal lining upon exposure to an inciting allergen (most commonly airborne dust mite fecal particles, cockroach residues, animal dander, molds, and pollens). T cells infiltrating the nasal mucosa are predominantly T helper 2 (Th2) in nature and release cytokines (e.g., interleukin [IL]-3, IL-4, IL-5, and IL-13) that promote IgE production by plasma cells. Cross linking of IgE bound to mast cells by allergens, in turn, triggers the release of mediators such as histamine and leukotrienes that are responsible for arteriolar dilation, increased vascular permeability, itching, rhinorrhea, mucous secretion, and smooth muscle contraction in the lung.^[24,25]^

Although there is no clear correlation between serum concentrations of specific IgG4 and clinical efficacy,^[26]^ Allergen-specific immunotherapy-induced specific IgG4 has been shown to act as a blocking antibody that competes with specific IgE for the binding of allergens affecting mast cells and basophil activity, and interfering with IgE-facilitated antigen presentation, thereby preventing the allergen-dependent activation of T cells.^[27,28]^ The blocking ability of specific IgG4 remains effect after completing the course of allergen-specific immunotherapy, despite the fact that levels of IgG4 have decreased.^[29]^ Specific IgG4 is thus an important immunological marker for SIT when objectively assessing the clinical performance of the treatment. It was reported that successful allergen-specific immunotherapy is always accompanied by a significant increase of specific IgG4 levels.^[30]^

Ten trials were included in our review. We found that YPFS was not inferior to Western medicine therapy of control group in terms of efficacy in pediatric AR patients. This might be the result of the trials having varying simple sizes and poor quality of experimental designation according to the GRADE methodology (Supplemental Digital Content Tables 3, 4, 5, 6 and 7). In these 10 studies, none applied validated questionnaires and scales that had been recommended by the 2015 Clinical Guideline.^[5]^ For example, the Quality of Life Questionnaire and visual analogue scale are used to evaluate the quality of life of AR patients. Visual analog scale is used to assess the severity of symptoms of AR. Daily life quality evaluations were also not performed in the included trials.

### 4.2 Limitation of our review

Despite all included studies using validated documents supporting diagnostic criteria and effectiveness assessment criteria, non-uniform diagnostic approaches and standards of efficacy evaluations might influence outcomes and results. It might be difficult to employ the same diagnosis and effectiveness assessment criteria for each trial, as these criteria varied in each study. Nevertheless, we employed the latest or most recently-published criteria.

### 4.3 Quality of evidence

All included studies were prospective, randomized, placebo-controlled studies. However, only 2 studies mentioned the method of randomization. No study stated whether the design was double-blinded. Therefore, there was a potential risk of measurement and implementation bias. No trial mentioned allocation concealment or any concealment method. It was not clear whether incomplete outcomes data were adequately addressed, as no trial reported drop-out rates. However, in Yang's study,^[19]^ there was information regarding dropouts from follow-up: only 11 patients in the treatment group and 6 patients in control group took part in the follow-up. Therefore, the risk of incomplete outcome data bias in this study was low risk, and in the others it was unclear. However, Yang did not perform an intention to treat analysis for cases of depigmentation in his follow-up. Fang's study^[15]^ mentioned their trial obtaining ethics association approval, and all patients signed the informed consent before the experiment. Therefore, selective reporting bias in this study was low risk, and the others are unclear. Xu^[18]^ made no reference to balance report of baseline, therefore other bias in this paper was high, and in the others it was low. Therefore, according to the GRADE system, the quality of evidence for the outcome measurement was low for 4 outcomes and very low for 5 outcomes.

### 4.4 Potential biases in the review process

Conclusions from this review were drawn from 10 included trials due to the absence of ongoing trials; there were a limited number of participants. More participants and high-quality design trials should be performed in the future. Another key issue was the timeframe for assessing outcome after treatment. The treatment course in these studies varied from 2 weeks to 3 months. In the control groups, several kinds of Western medications were designated “Western medicine therapy,” however, these interventions differed, with some being single medications and some being combinations. These critical differences might have direct influence on efficacy assessments and might be a factor leading to bias.

## 5 Conclusion

Current evidence suggests that YPFS cannot be recommended over Western medicine for the treatment of PAR. This may be due to poor quality study-design limitations of the included YPFS studies. Although for some adverse events, including headache, rash and dryness of the mouth, YPFS was relatively safe and reliable compared with Western medical therapy, a validated conclusion could not be drawn because only 5 studies were analyzed, and all included studies suffered from various methodological limitations. Therefore, larger sample sizes and rigorously-designed studies are necessary to determine conclusively a definitive association between YPFS and PAR.

### 5.1 Implications for practice

There is no support for the use of YPFS for the treatment of PAR.

### 5.2 Implications for research

There is an urgent need for double-blind, prospective, randomized, placebo-control trials of YPFS as a treatment for AR. Such studies should employ uniform and validated diagnostic criteria, efficacy evaluation criteria, recommended questionnaires, and measurement scales. Long-term follow-up effectiveness and safety studies for YPFS in PAR are necessary as well.

## Author contributions

Conceived and designed the experiments: YL, JZ, YZ, SQL. Performed the experiments: YL, ZJ, MLD, YPY, HYH. Analyzed the data: DL, TL, DZZ. Contributed reagents/materials/analysis tools: SQL, YPY, HMZ. Wrote the paper: YL, JZ, YZ.

**Conceptualization:** Yang Liu.

**Data curation:** YuZhao, Menglin Dai, Dazheng Zhang, Dan Lai, Tao Lu.

**Formal analysis:** Yu Zhao, Yang Liu.

**Funding acquisition:** Yu Zhao.

**Methodology:** Yu Zhao, Yepeng Yang, Dazheng Zhang, Dan Lai, Tao Lu.

**Software:** Xin rong Li, Dan Lai, Tao Lu.

**Writing – original draft:** Yong Liao, Juan Zhong.

## Supplementary Material

Supplemental Digital Content

## Supplementary Material

Supplemental Digital Content

## Supplementary Material

Supplemental Digital Content

## Supplementary Material

Supplemental Digital Content

## Supplementary Material

Supplemental Digital Content

## Supplementary Material

Supplemental Digital Content

## Supplementary Material

Supplemental Digital Content

## Supplementary Material

Supplemental Digital Content

## Supplementary Material

Supplemental Digital Content

## Supplementary Material

Supplemental Digital Content

## Supplementary Material

Supplemental Digital Content

## Supplementary Material

Supplemental Digital Content

## Supplementary Material

Supplemental Digital Content

## Supplementary Material

Supplemental Digital Content

## Supplementary Material

Supplemental Digital Content
