# Yu ping feng san for pediatric allergic rhinitis: A systematic review and meta-analysis of randomized controlled trials: Erratum

**DOI:** 10.1097/MD.0000000000025839

**Published:** 2021-05-14

In the article, “Yu ping feng san for pediatric allergic rhinitis: A systematic review and meta-analysis of randomized controlled trials”,^[1]^ which appeared in Volume 100, Issue 13 of *Medicine*, two authors have been added to the author list, Drs. Yong Liao and Dr. Yu Zhao. The author list, affiliations and contributions have been updated accordingly and now appear as:

Yong Liao, PHD^ab^, Juan Zhong, PhD^b,c^, Shuqin Liu, MD^c^, Menglin Dai, PhD^c^, Yang Liu, PhD^c^, Xinrong Li, PhD^c^, Yepeng Yang, PhD^c^, Dazheng Zhang, PhD^e,f,g^, Dan Lai, PhD^h^, Tao Lu, PhD^i^, Qinxiu Zhang, PhD^c,d^, Yu Zhao, MD, PHD^a^∗

^a^ Department of Otorhinolaryngology Head and Neck Surgery, West China Hospital of Sichuan University, Chengdu,

^b^ Department of Otorhinolaryngology Head and Neck Surgery, Minda Hospital of Hubei Minzu University, Enshi

^c^ Hospital of Chengdu University of Traditional Chinese Medicine, d School of Medical and Life Sciences/Reproductive and Women-Children Hospital, Chengdu

University of Traditional Chinese Medicine, e Chengdu University of Traditional Chinese Medicine, f Dujiangyan medical centre, g China qingcheng medical research

laboratory of Traditional Chinese Medicine, h Department of Otolaryngology Head and Neck Surgery, The Affiliated Hospital of Southwest Medical University, No. 25

TaiPing Street, Luzhou, Sichuan, China, i Otolaryngology and Head & Neck Surgery Department One, First Affiliated Hospital of Kunming Medical University, Kunming, Yunnan.

Conceived and designed the experiments: YL, JZ, YZ, SQL. Performed

the experiments: YL, ZJ, MLD, YPY, HYH. Analyzed the data: DL, TL, DZZ. Contributed

reagents/materials/analysis tools: SQL,YPY,HMZ. Wrote the paper: YL, JZ, YZ.

Conceptualization: Yang Liu.

Data curation: Yu Zhao, Menglin Dai, Dazheng Zhang, Dan Lai, Tao Lu.

Formal analysis: Yu Zhao, Yang Liu.

Funding acquisition: Yu Zhao.

Methodology: Yu Zhao, Yepeng Yang, Dazheng Zhang, Dan Lai, Tao Lu.

Software: Xin rong Li, Dan Lai, Tao Lu.

Writing – original draft: Yong Liao, Juan Zhong

Dr. Yu Zhao has been updated to the corresponding author.
