# Supplementary figures and images for: Yu ping feng san for pediatric allergic rhinitis: A systematic review and meta-analysis of randomized controlled trials
Source: Medicine (Baltimore). 2021 Apr 2;100(13):e24534. doi: 10.1097/MD.0000000000024534 (PMC8021384; doi:10.1097/MD.0000000000024534)

Fig. S1 Flow diagram of study selection process


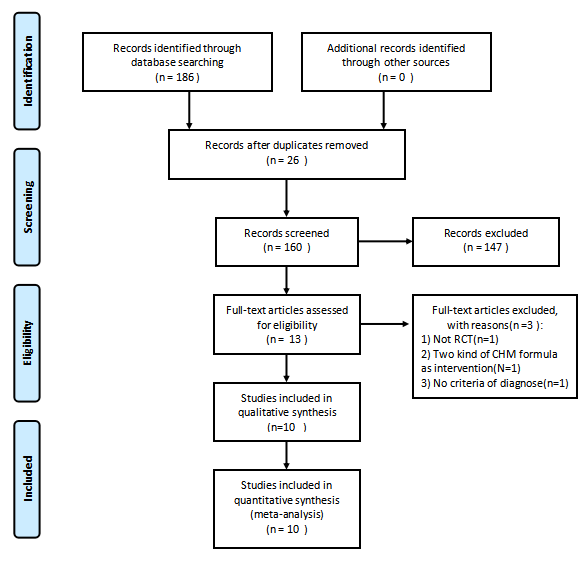

Supplement: Supplemental Digital Content [file medi-100-e24534-s001.docx]

Fig. S2 Risk of bias graph of authors' judgements regarding included studies


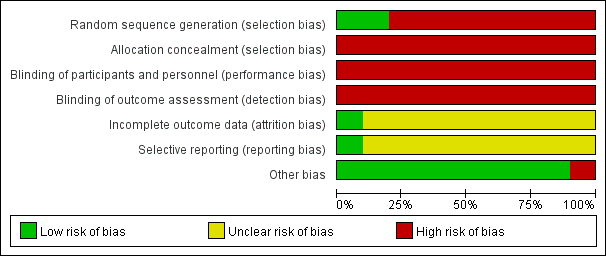

Supplement: Supplemental Digital Content [file medi-100-e24534-s002.docx]

Fig. S3. Risk of bias summary of authors' judgements regarding included studies


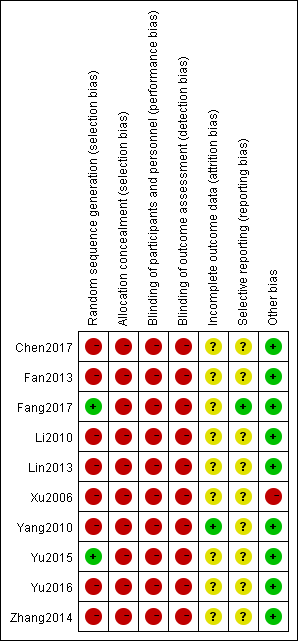

Supplement: Supplemental Digital Content [file medi-100-e24534-s003.docx]

Fig. S4. Efficacy of 8 RCTs of YPFS vs. Western medical therapy


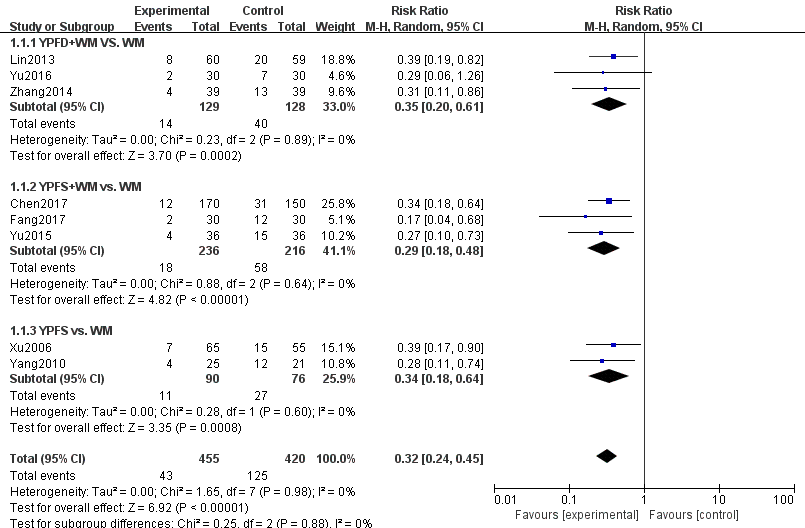

Supplement: Supplemental Digital Content [file medi-100-e24534-s004.docx]

Fig. S5. Variation of serum IgA in 3 RCTs


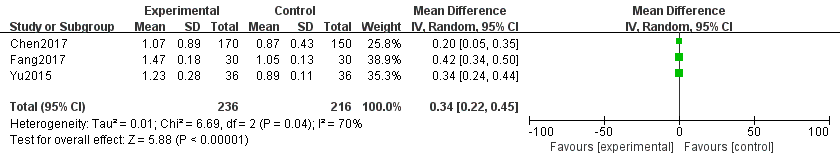

Supplement: Supplemental Digital Content [file medi-100-e24534-s005.docx]

Fig. S6. Variation of serum IgE in 3 RCTs


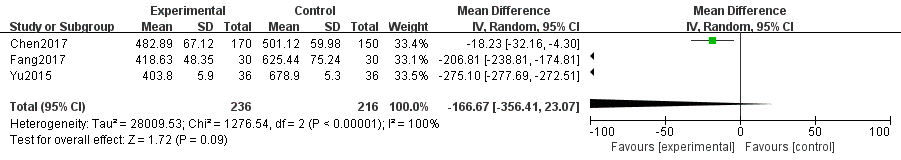

Supplement: Supplemental Digital Content [file medi-100-e24534-s006.docx]

Fig. S7. Variation of serum IgG in 3 RCTs


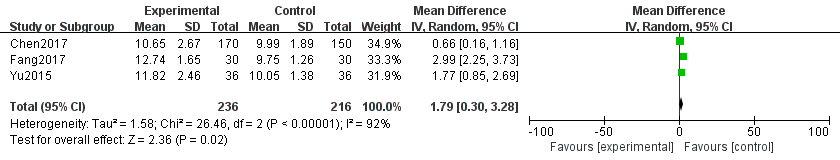

Supplement: Supplemental Digital Content [file medi-100-e24534-s007.docx]

Fig. S8. Safety of YPFS vs. Western medical therapy


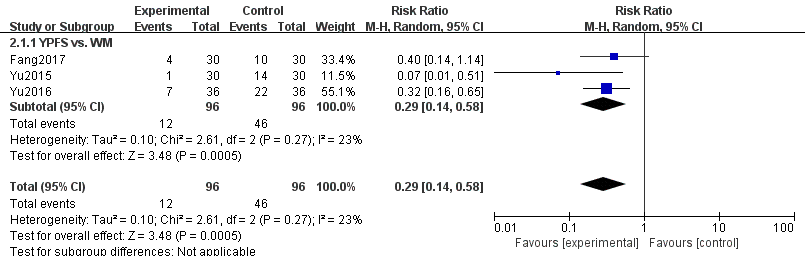

Supplement: Supplemental Digital Content [file medi-100-e24534-s008.docx]
