# Supplementary material for: Yu ping feng san for pediatric allergic rhinitis: A systematic review and meta-analysis of randomized controlled trials
Source: Medicine (Baltimore). 2021 Apr 2;100(13):e24534. doi: 10.1097/MD.0000000000024534 (PMC8021384; doi:10.1097/MD.0000000000024534)
Supplement: Supplemental Digital Content [file medi-100-e24534-s009.doc]

Table S1. Characteristics of included studies

| Trials | Sample size(E/C) | Gender(E/C) and age(yr) | Duration | Criteria of diagnose | Criteria of efficacy assessment | Interventions | | Period | Outcome measure | Balance report of baseline |
| --- | --- | --- | --- | --- | --- | --- | --- | --- | --- | --- |
|  |  |  |  |  |  | Experimental group | Control group |  |  |  |
| Zhang2014 | 78(39/39) | (45M:33F)/5-15 | 18months-5years | 1997 criteria | 1997 criteria | Modified Yu Ping Feng Decoction+WM | Fluticasone propionate nasal spray，Desloratadine Tablets | A month | effective rate | P＞0.05 |
| Li2010 | 128(64/64) | (37M:27F)/ (41M:23F) 3-13 | 3months-4years | 2001criteria | 2001criteria | Yu Ping Feng granular+WM | Budesonide nasal spray | 3 months | effective rate;adverse events | P＞0.05 |
| Fang2017 | 60(30/30) | (20M:10F)/ (20M:10F) 3-13 | 4months-5year | 2009criteria | 2009criteria | Yu Ping Feng granular+WM | Montelukast | A month | effective rate;adverse events;Serum IgA、IgG,IgE,IL-6,IL-17 and IL-23 level | P＞0.05 |
| Chen2017 | 320(170/150) | (88M:62F)/ (100M:70F) 4-14 | 5months-9years | 2009criteria | 2009criteria | Yu Ping Feng granular+WM | Singulair | A month | effective rate;IgA,IgG,IgE；PO2,PCO2 and WBC; | P＞0.05 |
| Yang2010 | 46(25/21) | (14M:11F)/ (12M:9F) 3-14 | 1months-6years | 1997criteria | 1997criteria | Yu Ping Feng granular | Loratadine Tablets | 2 months,2 months of follow-up | effective rate;adverse events;2 months recurrence rate | P＞0.05 |
| Fan2013 | 66(36/30) | (19M:17F)/ (12M:18F) 3-13 | 1months-5years | 1997criteria | 1997criteria | Yu Ping Feng granular+WM | Loratadine Tablets dry suspension | 14days | effective rate;adverse events | P＞0.05 |
| Xu2006 | 120(65/55) | (33M:32F)/ (26M:29F) 8months-12 | 6months-9years | 1997criteria | 1997criteria | Yu Ping Feng granular | DINK | 3months | effective rate | not mentioned |
| Yu2016 | 60（30/30） | (18M:12F)/ (14M:16F) 3-14 | mean 13.32 months | 1994criteria | 1994criteria | Modified Yu Ping Feng Decoction+WM | Oxymetazoline hydrochloride nasal spray,Cetirizine | A month | effective rate;adverse events;Olfactory recovery time,Edema disappearance time,degree of satisfaction | P＞0.05 |
| Yu2015 | 72（36/36) | (19M:17F)/ (16M:20F) 5-15 | 6months-10years | 1997 criteria | 1997criteria | Yu Ping Feng granular+WM | Singulair | A month | effective rate;adverse events;Clinical symptom score;Clinical signs score;serum IgA,IgG,IgE | P＞0.05 |
| Lin2013 | 119(60/59) | (38M:22F)/ (36M:23F) 4-14 | Not mentioned | 2010 criteria | 2010 criteria | Modified Yu Ping Feng Decoction+WM | Budesonide nasal aerosol, Loratadine Tablets | 8 weeks | effective rate;1 months recurrence rate | P＞0.05 |
