# Supplementary material for: Yu ping feng san for pediatric allergic rhinitis: A systematic review and meta-analysis of randomized controlled trials
Source: Medicine (Baltimore). 2021 Apr 2;100(13):e24534. doi: 10.1097/MD.0000000000024534 (PMC8021384; doi:10.1097/MD.0000000000024534)
Supplement: Supplemental Digital Content [file medi-100-e24534-s010.doc]

Table S2. Characteristics of excluded studies

| Trials | Sample size(E/C) | Gender(E/C) and age(yr) | Duration | Criteria of diagnose | Criteria of efficacy assessment | Interventions | | Period | Outcome measure | Balance report of baseline | Why removed |
| --- | --- | --- | --- | --- | --- | --- | --- | --- | --- | --- | --- |
|  |  |  |  |  |  | Experimental group | Control group |  |  |  |  |
| Du2017 | 108(54/54) | (30M:24F)/ (32M:22F) 2-13 | 1-52months | Not mentioned | Not mentioned | Yu Ping Feng granular | Loratadine Tablets | A month | effective rate;CD3,CD4,CD8;CRP；Qulity of life | P＞0.05 | No diagnose criteria |
| Ye2014 | 119(61/58) | (32M:29F)/ (31M:27F) 2-13 | 4w-9months | 1997 criteria | 1997 criteria | Yu Ping Feng granular+Montelukast | Montelukast | A month | effective rate；one year of recurrence rate | P＞0.05 | se-mi RCT |
| Wu2014 | 106(56/50) | (38M:18F)/ (35M:15F) 6-13 | 3months-1year | 2001criteria | 2001criteria | Yu Ping Feng granular+Cang er zi granular+Budesonide Nasal Spray | Budesonide Nasal Spray | 3 months | effective rate; adverse events | P＞0.05 | Two herbal medicine formulas combined as intervention |
