# Supplementary material for: Yu ping feng san for pediatric allergic rhinitis: A systematic review and meta-analysis of randomized controlled trials
Source: Medicine (Baltimore). 2021 Apr 2;100(13):e24534. doi: 10.1097/MD.0000000000024534 (PMC8021384; doi:10.1097/MD.0000000000024534)
Supplement: Supplemental Digital Content [file medi-100-e24534-s012.doc]

Supplemental Digital Content Table3 Quality of evidence for outcome measure of efficacy

| **Quality assessment** | | | | | | | **Summary of findings** | | | | | **Importance** |
| --- | --- | --- | --- | --- | --- | --- | --- | --- | --- | --- | --- | --- |
|  |  |  |  |  |  |  | **No of patients** | | **Effect** | | **Quality** |  |
| **No of studies** | **Design** | **Limitations** | **Inconsistency** | **Indirectness** | **Imprecision** | **Other considerations** | **effective rate** | **control** | **Relative (95% CI)** | **Absolute** |  |  |
| **YPFS vs. WM** | | | | | | | | | | | | |
| 8 | randomised trials^1^ | serious^1,2^ | serious^3^ | no serious indirectness^4^ | very serious^5^ | none^6^ | 43/455 (9.5%) | 125/420 (29.8%) | RR 0.32 (0.24 to 0.45) | 202 fewer per 1000 (from 164 fewer to 226 fewer) | ⊕OOO VERY LOW | IMPORTANT^7^ |
|  |  |  |  |  |  |  |  | 33.6% |  | 228 fewer per 1000 (from 185 fewer to 255 fewer) |  |  |
| **YPFS vs. WM - YPFD+WM VS. WM** | | | | | | | | | | | | |
| 3 | randomised trials^1^ | serious^1^ | serious^3^ | no serious indirectness^4^ | very serious^5^ | none^6^ | 14/129 (10.9%) | 40/128 (31.3%) | RR 0.35 (0.2 to 0.61) | 203 fewer per 1000 (from 122 fewer to 250 fewer) | ⊕OOO VERY LOW | IMPORTANT^2^ |
|  |  |  |  |  |  |  |  | 33.3% |  | 216 fewer per 1000 (from 130 fewer to 266 fewer) |  |  |
| **YPFS vs. WM - YPFS+WM vs. WM** | | | | | | | | | | | | |
| 3 | randomised trials^1^ | no serious limitations^1^ | serious^3^ | no serious indirectness^4^ | very serious^5^ | none^6^ | 18/236 (7.6%) | 58/216 (26.9%) | RR 0.29 (0.18 to 0.48) | 191 fewer per 1000 (from 140 fewer to 220 fewer) | ⊕OOO VERY LOW | IMPORTANT^7^ |
|  |  |  |  |  |  |  |  | 40% |  | 284 fewer per 1000 (from 208 fewer to 328 fewer) |  |  |
| **YPFS vs. WM - YPFS vs. WM** | | | | | | | | | | | | |
| 2 | randomised trials^1^ | serious^1^ | serious^3^ | no serious indirectness^4^ | very serious^5^ | reporting bias^6^ | 11/90 (12.2%) | 27/76 (35.5%) | RR 0.34 (0.18 to 0.64) | 234 fewer per 1000 (from 128 fewer to 291 fewer) | ⊕OOO VERY LOW | CRITICAL^7^ |
|  |  |  |  |  |  |  |  | 42.2% |  | 279 fewer per 1000 (from 152 fewer to 346 fewer) |  |  |

^1^ Some studies had a high risk of bias due to their methodology
^2^ The study had performance bias and detection bias
^3^ Total number of events is less than 300
^4^ All studies were from China
^5^ Only one study or two studies
^6^ Further research is needed
^7^ Uncertain about the estimate
