# Supplementary material for: Yu ping feng san for pediatric allergic rhinitis: A systematic review and meta-analysis of randomized controlled trials
Source: Medicine (Baltimore). 2021 Apr 2;100(13):e24534. doi: 10.1097/MD.0000000000024534 (PMC8021384; doi:10.1097/MD.0000000000024534)
Supplement: Supplemental Digital Content [file medi-100-e24534-s013.doc]

Supplemental Digital Content Table4 Quality of evidence for outcome measures of safety

| **Quality assessment** | | | | | | | **Summary of findings** | | | | | **Importance** |
| --- | --- | --- | --- | --- | --- | --- | --- | --- | --- | --- | --- | --- |
|  |  |  |  |  |  |  | **No of patients** | | **Effect** | | **Quality** |  |
| **No of studies** | **Design** | **Limitations** | **Inconsistency** | **Indirectness** | **Imprecision** | **Other considerations** | **adverse events** | **control** | **Relative (95% CI)** | **Absolute** |  |  |
| **adverse events** | | | | | | | | | | | | |
| 3 | randomised trials^1^ | no serious limitations^1^ | no serious inconsistency^2^ | no serious indirectness^3^ | very serious^4^ | none^5^ | 12/96 (12.5%) | 46/96 (47.9%) | RR 0.29 (0.14 to 0.58) | 340 fewer per 1000 (from 201 fewer to 412 fewer) | ⊕⊕OO LOW | CRITICAL^6^ |
|  |  |  |  |  |  |  |  | 46.7% |  | 332 fewer per 1000 (from 196 fewer to 402 fewer) |  |  |
| **adverse events - YPFS vs. WM** | | | | | | | | | | | | |
| 3 | randomised trials^1^ | no serious limitations^1^ | no serious inconsistency^2^ | no serious indirectness^3^ | very serious^4^ | none^5^ | 12/96 (12.5%) | 46/96 (47.9%) | RR 0.29 (0.14 to 0.58) | 340 fewer per 1000 (from 201 fewer to 412 fewer) | ⊕⊕OO LOW | CRITICAL^6^ |
|  |  |  |  |  |  |  |  | 46.7% |  | 332 fewer per 1000 (from 196 fewer to 402 fewer) |  |  |

^1^ Some studies had a high risk of bias due to their methodology
^2^ The study had performance bias and detection bias
^3^ Total number of events is less than 300
^4^ All studies were from China
^5^ Only one study or two studies
^6^ Further research is needed
^7^ Uncertain about the estimate
